# Can fundus features tell us something about 3D eye shape?

**DOI:** 10.1111/opo.13454

**Published:** 2025-01-24

**Authors:** Fabian Yii, Niall C. Strang, Samuel Gibbon, Tom J. MacGillivray

**Affiliations:** ^1^ Robert O Curle Ophthalmology Suite, Institute for Regeneration and Repair The University of Edinburgh Edinburgh UK; ^2^ Centre for Clinical Brain Sciences The University of Edinburgh Edinburgh UK; ^3^ Department of Vision Sciences Glasgow Caledonian University Glasgow UK

**Keywords:** eye shape, fundus, magnetic resonance imaging, myopia, refractive error, retina

## Abstract

**Purpose:**

To determine whether imaging features derived from fundus photographs contain 3D eye shape information beyond that available from spherical equivalent refraction (SER).

**Methods:**

We analysed 99 eyes of 68 normal adults in the UK Biobank. An ellipsoid was fitted to the entire volume of each posterior eye (vitreous chamber without the lens)—segmented from magnetic resonance imaging of the brain. Asphericity was computed based on the semidiameters of the ellipsoid's axes to describe posterior eye shape along the horizontal (temporal–nasal) and vertical (superior–inferior) meridians, while volume was calculated as the total number of foreground voxels. Mixed‐effects linear regression models were used to test the association of SER with asphericity and volume, controlling for age and sex. Then, the association between various fundus features and asphericity was tested—both before and after controlling for SER, age and sex.

**Results:**

Posterior eyes were generally oblate (asphericity > 0), but the degree of oblateness reduced as SER decreased, with the shape tending towards prolateness in high myopia. Neither sex nor age influenced asphericity. However, males had larger posterior eyes on average (this difference disappeared after height was additionally controlled for). Optic disc (OD) orientation, OD‐fovea angle, vessel tortuosity, vessel fractal dimension and central retinal arteriolar or venular equivalent (CRAE or CRVE) showed significant univariable associations with asphericity along at least one meridian. After controlling for SER, age and sex, a more negative OD‐fovea angle (larger OD‐fovea angular separation) remained significantly associated with reduced horizontal oblateness (*p* = 0.01). Similarly, decreasing CRAE (narrower arterioles) remained significantly associated with reduced oblateness along both the horizontal (*p* = 0.04) and vertical (*p* < 0.01) meridians.

**Conclusions:**

Variations in OD‐fovea angle and CRAE are associated with differences in ocular asphericity—even in eyes with similar SER—suggesting that fundus imaging provides eye shape information beyond what is available from refractive error alone.

**Key points**

- In contrast to the ‘one‐dimensional’ nature of refractive error—which is essentially an *on‐axis* (along the visual axis) summary of ocular dimensions—morphological and geometrical properties of the fundus reflect changes *across* the posterior pole.
- Variations in optic disc–fovea angle and retinal vessel width may indicate differences in three‐dimensional eye shape, even among eyes with similar on‐axis refraction.
- Some fundus features may, therefore, offer additional prognostic value for conditions in which eye shape is clinically relevant.

## INTRODUCTION

Research assessing ocular dimensions along the visual axis has long demonstrated excessive vitreous chamber elongation in myopia.^1^ Moving beyond this exclusively on‐axis characterisation of ocular dimensions, more recent studies have additionally incorporated measurements along other dimensions using magnetic resonance imaging (MRI).^2^, ^3^, ^4^, ^5^, ^6^, ^7^ Findings from these studies suggest that the eye becomes less oblate (or more prolate) in higher myopia, with the posterior segment steepening at a higher rate centrally than peripherally, giving rise to a more pointed or curved posterior pole appearance. Indirect estimates of posterior eye shape based on peripheral axial length and refraction also suggest that the central retina steepens more than the peripheral retina in myopia.^8^, ^9^

The extent to which the posterior segment of the eye deviates from a sphere‐like shape has clinical implications for myopia. A more prolate (or less oblate) eye shape—based on indirect estimates using peripheral refraction—has been postulated to be a risk factor for faster axial growth or myopic shift in central refraction during childhood,^10^, ^11^ although conflicting findings exist in the literature.^12^, ^13^, ^14^ Several studies have also found a connection between posterior eye shape and the risk of myopic complications. Using optical coherence tomography (OCT), a more pointed posterior pole has been linked to an increased occurrence of myopic traction maculopathy (MTM) in highly myopic adults.^15^ Using MRI, a smaller angular separation between two points placed equidistant from either side of the base of the posterior pole, suggesting a more pointed posterior pole, has also been found to be associated with a higher frequency of MTM and myopic maculopathy.^16^ Further evidence linking the severity of myopic maculopathy to MRI‐derived eye shape has similarly been found elsewhere.^17^

The relevance of eye shape to the risk of myopia and its pathological sequelae suggests that it may be a valuable prognostic biomarker in clinical practice. In our recent work analysing over 30,000 fundus photographs in the UK Biobank, a wide range of retinal parameters were found to vary in a highly nonlinear fashion across refractive error,^18^ tying in with the observation that the odds of myopic complications increase exponentially, or quasi‐exponentially, with increasing myopia.^19^ Variations in eye shape patterns across refractive error were postulated to be one important reason behind this non‐linearity. For example, in myopia, an increasingly non‐uniform expansion of the posterior eye (increasing tendency towards prolateness) may result in increasing rates of mechanical stretching at the posterior pole and, consequently, increasing rates of fundus changes as myopia increases.

Unlike the ‘one‐dimensional’ nature of refractive error—which is essentially an *on‐axis* summary of ocular dimensions—variations in retinal geometry/morphology and spatial relationships between fundus landmarks reflect changes *across* the posterior pole. This implies that fundus features may contain additional ‘off‐axis’ information related to retinal or posterior eye shape, over and above what is available from refractive error alone. To test this hypothesis, we examined the association between fundus features and MRI‐derived posterior eye shape, while controlling for refractive error.

## METHODS

### Participants

Study participants were derived from the UK Biobank, a major biomedical database described previously.^20^ Briefly, around half a million community‐dwelling residents from across the UK participated in an extensive range of assessments between 2006 and 2010. A subset of these (around 68,000) additionally underwent a standardised ophthalmic assessment from 2009 to 2010. Around 20,000 participants attended a follow‐up visit from August 2012 to June 2013 (hereafter referred to as the retinal imaging visit), during which the same assessments—including 45° macula‐centred colour fundus photography using the Topcon 3D OCT‐1000 Mark II (Topcon Corp., topconhealthcare.com), visual acuity (VA) evaluation using the Precision Vision digital logMAR chart (Precision Vision, precision‐vision.com), auto‐refraction/keratometry using the Tomey RC‐5000 (Tomey, tomey.com) and anthropometric measurements using standard scales (Seca GmbH, seca.com)—were carried out.

Structural brain imaging using MRI was subsequently introduced in 2014.^21^ Of those invited to undergo MRI, 891 phakic participants were identified to have a reasonably short lapse of time (<2 years) between the retinal imaging and MRI visits. After removing eyes with poor VA (logMAR > 0.00), missing VA measurements or missing auto‐refraction/keratometry data, 1029 eyes of 650 participants remained. Twenty‐nine eyes with a history of chorioretinal disease, globe/scleral disease or strabismus identified based on linked healthcare data^22^ were further removed. Both eyes of participants with hypertension or diabetes were also excluded, following which 679 eyes of 427 participants remained. Of these, 441 eyes of 303 participants had adequate‐quality colour fundus photographs (Data S2). A large proportion of the remaining eyes had unusable MRI scans characterised by the absence of a significant portion of the eye (Data S2), as the scans in the UK Biobank were defaced to protect participants' anonymity (voxels in the face and ear regions were masked).^23^ After excluding these, 99 eyes of 68 participants were finally included in the study.

### MRI acquisition and analysis

**FIGURE 1:**
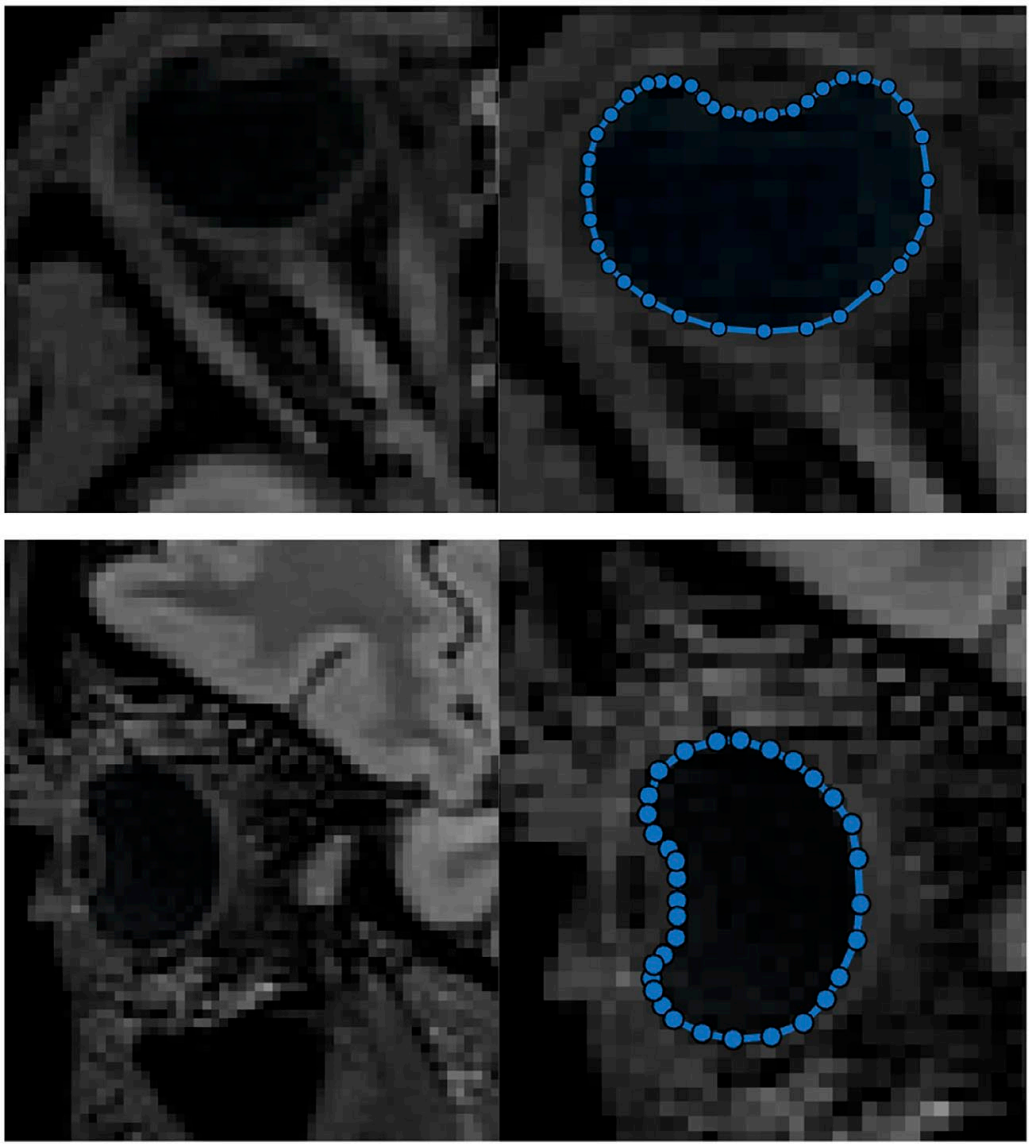
A zoomed‐in view of a magnetic resonance imaging slice in the transverse plane (top) and sagittal plane (bottom). The posterior segment of the eye—defined as the vitreous chamber (which appears hypointense relative to the ocular and lenticular walls) excluding the crystalline lens—is segmented by tracing its boundary using waypoints (blue‐filled circles on the right).

Whole‐brain T2‐weighted fluid‐attenuated inversion recovery (FALIR) scans with voxel dimensions of 1.05 mm × 1 mm × 1 mm (192 × 256 × 256 matrix) were acquired with a 3 T Siemens Skyra MRI scanner (Siemens Healthineers, siemens‐healthineers.com) using a standard 32‐channel head coil. A description of the MRI acquisition protocol is available elsewhere.^21^ The acquired scans were converted to Neuroimaging Informatics Technology Initiative format^24^ for subsequent analysis. On a T2‐weighted FLAIR scan, the vitreous body appears hypointense (darker) relative to the surrounding structures, making identification of the posterior segment/eye, defined as the vitreous chamber without the crystalline lens, a straightforward task (Figure 1).

A single operator (FY) manually segmented the posterior segment of each eye from MRI slices that contained the foreground pixels, using the Medical Image Labeler application in MATLAB (MathWorks, mathworks.com).^25^ The application provided an interface for interactive cursor‐based segmentation of regions of interest by allowing their boundaries to be traced using waypoints. The same operator repeated the segmentation at least 1 month later for 20 eyes selected using stratified sampling, where five eyes were *randomly* sampled from each quartile of spherical equivalent refraction (SER). A second annotator (SG) also manually segmented the same 20 eyes. Intra‐observer (FY's initial vs. repeated segmentation) and inter‐observer (FY's initial segmentation vs. SG's segmentation) repeatability was assessed using the Dice score, where a value of 1 indicated a perfect overlap:

$$\frac{\text{Number of common segmented pixels}\times 2}{\text{Total number of segmented pixels}}$$

**FIGURE 2:**
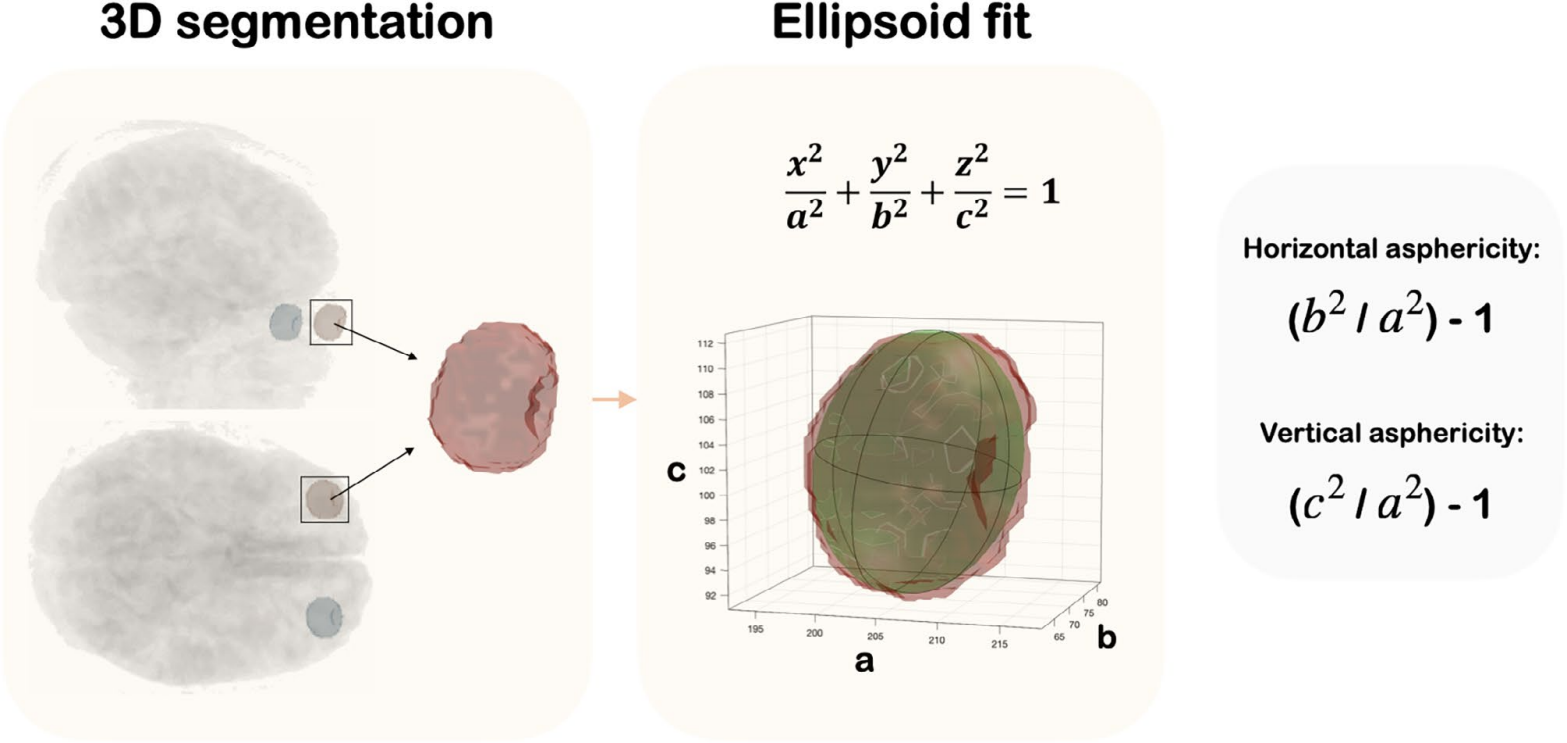
Derivation of horizontal and vertical asphericity from a three‐dimensional (3D) ellipsoid fitted to the posterior segment of a left eye, manually segmented from the magnetic resonance imaging scan of the brain. Anterior–posterior, nasal–temporal and inferior–superior semidiameters are denoted by a, b and c, respectively.

A 3D *ellipsoid* was fitted to the entire *volume* of each segmented posterior eye (Figure 2) using principal component analysis (PCA),^26^ where the axes of the fitted ellipsoid were represented by the eigenvectors of the covariance matrix of the data. PCA allows for ellipsoidal rotation during model fitting, which is advantageous because it need not assume that the eye is in perfect primary gaze or that the vertex of the posterior eye is fixed. The goodness of fit was assessed by calculating the root mean square (RMS) error between the fitted ellipsoid and the segmentation. To ensure the ellipsoid was an appropriate model for all eyes, irrespective of refractive error, each fitted ellipsoid was visually inspected alongside the segmentation overlay. The association between SER and RMS error was also examined to determine if SER had any unwanted influence on the goodness of fit.

An ellipsoid had the following general form:

$$\frac{x^{2}}{a^{2}}+\frac{y^{2}}{b^{2}}+\frac{z^{2}}{c^{2}}=1$$

Where *a*, *b* and *c* represented the semi‐diameters, in mm, along the *x* (anterior–posterior), *y* (nasal–temporal) and *z* (inferior–superior) axes, respectively. The degree of asphericity of the fitted ellipsoid, related to the prolateness or oblateness of the posterior eye, was calculated as $\frac{b^{2}}{a^{2}}-1$ along the horizontal (nasal–temporal) meridian and $\frac{c^{2}}{a^{2}}-1$ along the vertical (inferior–superior) meridian. An asphericity value of zero along a given meridian indicated that the posterior eye shape (along that meridian) conformed to a perfect spherical shape. Conversely, a value different from zero suggested some degree of deviation from a spherical shape, where a negative value (relatively *long* anterior–posterior axis) corresponded to a prolate shape, while a positive value (relatively *short* anterior–posterior axis) corresponded to an oblate shape. The volume of the posterior eye (in mm^3^) was calculated as the total number of foreground voxels.

### Fundus features

**FIGURE 3:**
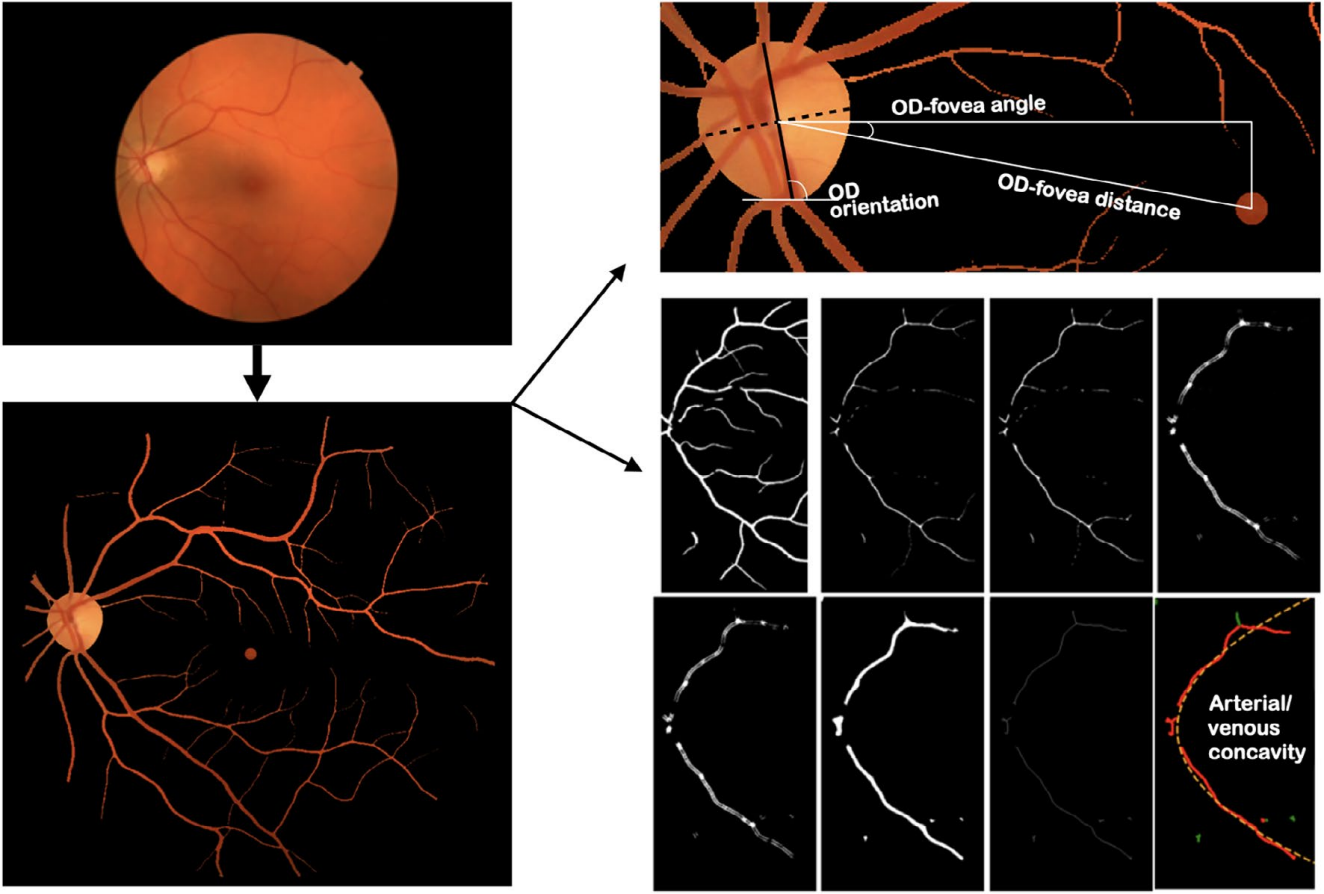
Overview of selected fundus features representing various measures of optic disc (OD), foveal and retinal vascular morphology or geometry, as listed in Table 1 and detailed in Yii et al.^18^

Twelve fundus features were derived using methods detailed in our recent work,^18^ except that optic disc (OD) segmentation and foveal localisation were performed manually by an experienced operator (FY), who was masked to participant characteristics during this process. Readers are referred to our previous publications for a description of this process, which demonstrated high intra‐operator (FY) and inter‐operator (FY and SG) repeatability.^27^, ^28^ Manual OD segmentation and foveal localisation were preferred over fully automated approaches in this study because both tasks were straightforward—and the dataset was small—thus allowing for direct quality control when performed manually. An overview of the fundus features is provided in Table 1 and illustrated in Figure 3. The influence of ocular magnification on dimensional metrics was accounted for using the approach proposed by Garway‐Heath et al.^29^ and further detailed in Data S1.

**TABLE 1:** Description of each fundus imaging feature.

Fundus feature	Description
OD‐fovea distance (pixel)	Euclidian distance between OD centre and fovea. Larger values indicate greater distance
OD‐fovea angle (**°**)	Angle between horizontal axis of the image passing through the OD centre and a straight line from the OD centre to the fovea. Smaller (more negative) values indicate greater angular separation
OD area (pixel^2^)	Area computed using the standard formula for an ellipse. Larger values indicate a larger area
OD orientation (**°**)	Angle between horizontal axis of the image passing through the OD centre and major axis of the disc. Larger values indicate a more vertical or less oblique/horizontal orientation
OD ovality	Ratio of OD major axis length to OD minor axis length. Larger values indicate a less circular, more oval appearance
CRAE (pixel)	Reflects the calibre of central retinal arterioles. Larger values indicate a greater calibre
CRVE (pixel)	Reflects the calibre of central retinal venules. Larger values indicate a greater calibre
Vessel tortuosity	Reflects the tortuosity of both retinal arterioles and venules. Larger values indicate increased tortuosity
Vessel FD	Reflects branching complexity of both retinal arterioles and venules. Larger values indicate increased complexity
Arterial concavity	Reflects the parabolic course of the major temporal arterial arcade. Larger values indicate increased concavity towards the fovea
Venous concavity	Reflects the parabolic course of the major temporal venous arcade. Larger values indicate increased concavity towards the fovea

Abbreviations: CRAE, central retinal arteriolar equivalent; CRVE, central retinal venular equivalent; FD, fractal dimension; OD, optic disc.

### Statistical analysis

The age‐ and sex‐adjusted association of SER (independent variable) was first assessed with horizontal/vertical asphericity and posterior eye volume. Then, univariable association between each fundus feature (independent variable) and horizontal/vertical asphericity was explored before including SER, age and sex as covariates to identify fundus features *independently* associated with eye shape. All non‐MRI variables, including fundus features, SER and age, were specific to the retinal imaging visit as detailed above. All continuous independent variables and covariates were standardised to have zero mean and unit variance. Linear mixed‐effects (random intercept) models were fitted in all instances, with individuals as random effects to account for inter‐eye correlation, using the *lmerTest* package in R version 4.2.2 (R Core Team 2022, r‐project.org).^30^ The source code is available at github.com/fyii200/3DeyeShapeRefractiveError.

## RESULTS

The majority (*n* = 65) of the 68 study participants self‐reported as ‘British’, ‘Irish’, ‘White’ or ‘Other white background’, with 49 being female. The mean ± SD (range) age, SER and lapse of time between the retinal imaging and MRI visits were 54.5 ± 7.7 (45–71) years, −0.26 ± 2.46 (−11.69 to 5.31) D and 1.7 ± 0.3 (1–2) years, respectively. Defining myopia as SER < −0.50 D, 26 eyes of 19 participants were myopic.

**FIGURE 4:**
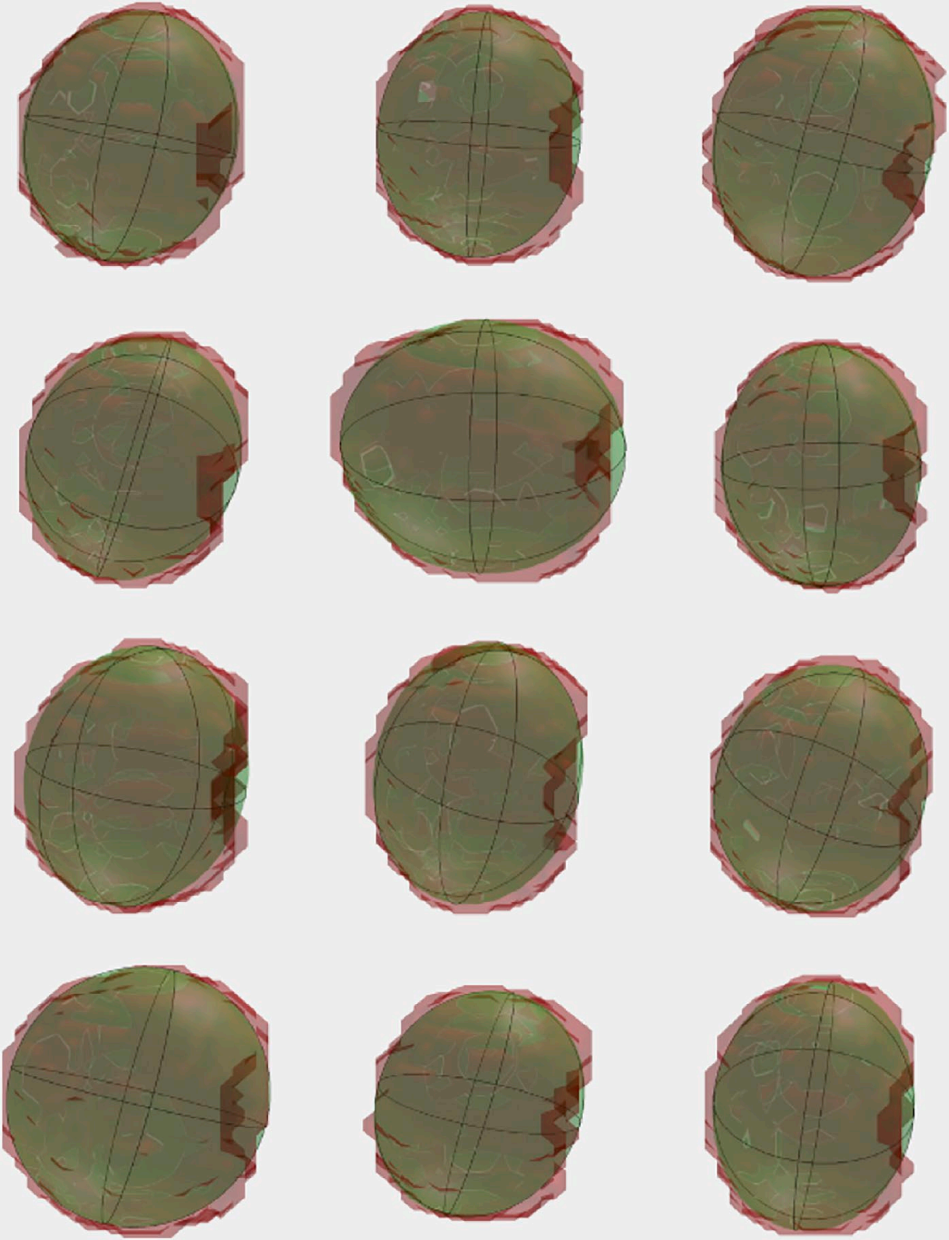
Examples of segmented posterior eyes (pink/maroon overlays) along with the best‐fitting ellipsoids (green) in the sagittal plane, demonstrating excellent goodness of fit. A visualisation of the goodness of fit, with the eyes rotating around the vertical axis, is available as Video S1 and at github.com/fyii200/3DeyeShapeRefractiveError.

Manual segmentation of the posterior eyes was found to be highly repeatable, with a mean intra‐ and inter‐observer Dice score of 0.91 and 0.88, respectively. This high repeatability was evident even for the least repeatable segmentation, where the Dice score was 0.86 for the same observer and 0.80 between observers. Similarly, the intraclass correlation coefficients (ICCs) for key ellipsoidal parameters derived from FY's initial segmentation and FY's repeated segmentation were high: 0.86 (anterior–posterior semidiameter), 0.97 (nasal–temporal semidiameter) and 0.88 (inferior–superior semidiameter). The ICCs comparing FY's initial segmentation to SG's segmentation were also high for these parameters: 0.86, 0.84 and 0.89. Visual inspection of each fitted ellipsoid (representative examples shown in Figure 4 and Video S1) indicated excellent goodness of fit for all eyes. The mean RMS error was smaller (0.50 mm) than the MRI's spatial resolution (~1 mm). Additionally, no association between SER and RMS error was found (*p* = 0.72; coefficient of determination = 0.002).

**FIGURE 5:**
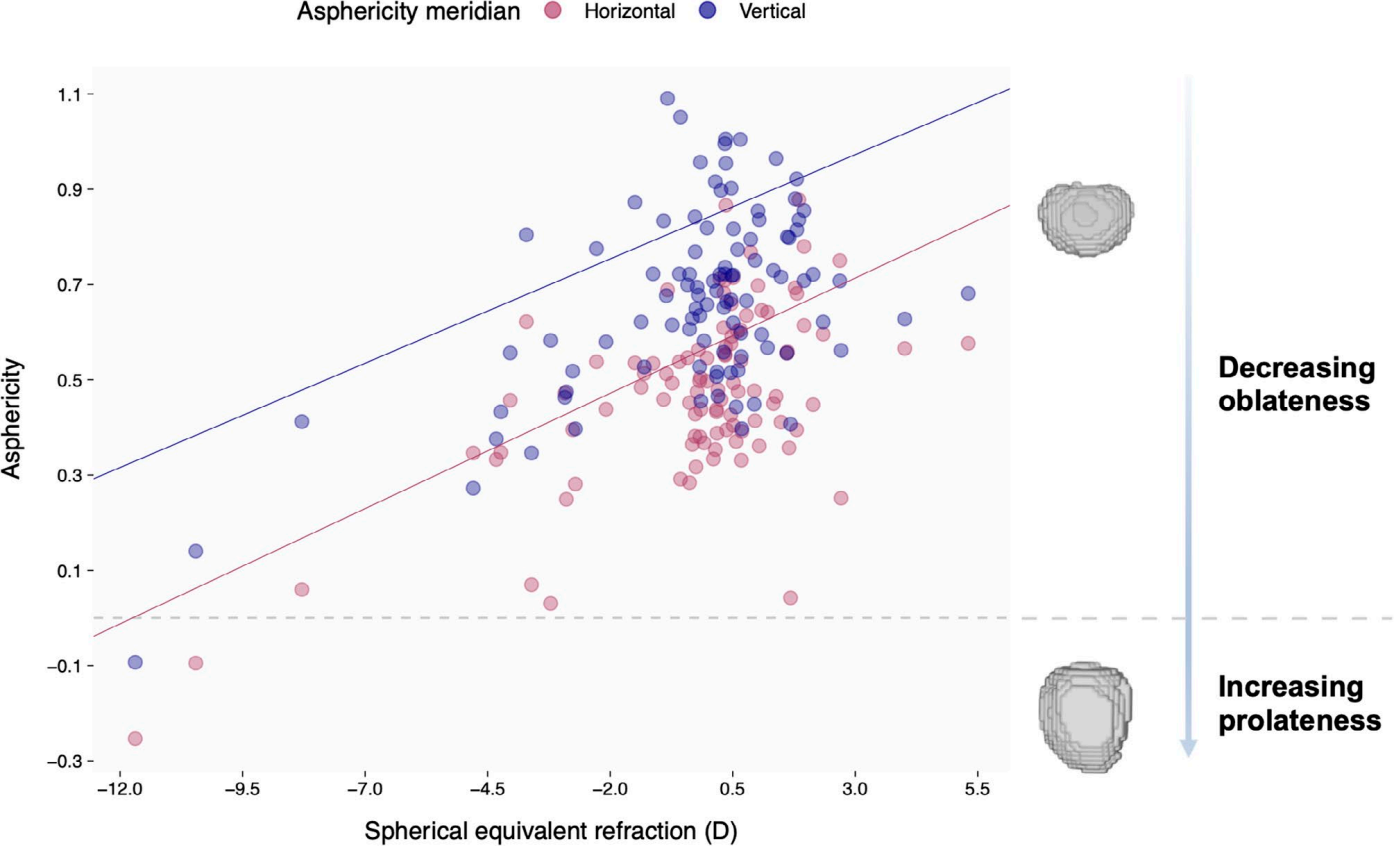
Scatterplot with regression lines (adjusted for age and sex) illustrating the positive association between SER and asphericity along the horizontal (red points) and vertical (blue points) meridians. Dotted horizontal line represents a perfect spherical shape (zero asphericity); values below this line indicate a prolate shape, while values above indicate an oblate shape. Examples of posterior eyes with different degrees of horizontal asphericity are displayed on the right side of the plot.

Most posterior eyes (97 of 99) in this dataset had a non‐prolate shape (asphericity ≥0 along both meridians), even in the presence of myopia (only two myopic eyes were prolate). The vertical meridian also appeared less oblate—on average—than the horizontal meridian (Figure 5). That said, decreasing SER was associated with reduced oblateness (less positive asphericity) along either meridian (Figure 5), while both age and sex had no influence on asphericity (Table 2). The mean (SD) posterior eye volume was 4433 (715) mm,^3^ with an increase of 240 mm^3^ for every 2.50 D increase in SER in the myopic direction (Table 2). Compared with females, the posterior eye was, on average, 469 mm^3^ larger in males. However, this sex difference was attributable to the confounding effect of (standing) height, as the significant association between male sex and posterior eye volume disappeared (β: 217, 95% CI: −271 to 706, *p* = 0.40) after height was controlled for.

**TABLE 2:** Association among spherical equivalent refraction (SER, independent variable) and MRI‐derived parameters, controlling for age and sex.

	Horizontal asphericity		Vertical asphericity		Posterior eye volume	
	β [95% CI]	*p*	β (95% CI)	*p*	β (95% CI)	*p*
SER (per 2.5 D)	0.12 [0.08 to 0.15]	<0.001	0.11 [0.07 to 0.15]	<0.001	−240 [−382 to −98]	<0.01
Age (per 7.7 years)	−0.01 [−0.04 to 0.02]	0.57	−0.02 [−0.06 to 0.02]	0.26	−8 [−155 to 140]	0.92
Male	−0.02 [−0.10 to 0.06]	0.61	−0.0003 [−0.08 to 0.08]	0.99	469 [137 to 801]	0.01

Abbreviations: MRI, magnetic resonance imaging; β, standardised beta coefficient.

Several fundus features showed significant *univariable* associations with posterior eye shape (Table 3). A decrease in vessel fractal dimension (FD)—indicating lower vascular complexity—and a reduction in central retinal arteriolar or venular equivalent (CRAE or CRVE) were associated with reduced oblateness along both the horizontal and vertical meridians. Additionally, a more negative OD‐fovea angle (indicating greater OD‐fovea angular separation) was associated with reduced oblateness along the horizontal meridian, while a more obliquely orientated OD and less tortuous retinal vasculature were associated with reduced oblateness along the vertical meridian. After controlling for SER, age and sex, a more negative OD‐fovea angle and smaller CRAE remained significantly associated with a decrease in horizontal oblateness. Likewise, a positive association between CRAE and asphericity along the vertical meridian was still evident after covariate adjustment.

**TABLE 3:** Association between each fundus feature (independent variable) and ocular asphericity, with and without adjustment for spherical equivalent refraction, age and sex.

	Horizontal asphericity				Vertical asphericity			
	β [95% CI]	*p*	β (95% CI)^a^	*p* ^a^	β (95% CI)	*p*	β (95% CI)^a^	*p* ^a^
OD‐fovea distance	0.01 [−0.03 to 0.05]	0.59	0.02 [−0.02 to 0.05]	0.33	−0.01 [−0.05 to 0.03]	0.54	−0.01 [−0.05 to 0.02]	0.46
OD orientation	0.02 [−0.01 to 0.05]	0.19	0.003 [−0.02 to 0.03]	0.84	0.04 [0.003 to 0.08]	0.03	0.02 [−0.01 to 0.05]	0.27
OD ovality	−0.01 [−0.04 to 0.02]	0.61	−0.01 [−0.03 to 0.02]	0.53	−0.01 [−0.04 to 0.03]	0.76	−0.005 [−0.04 to 0.03]	0.78
OD area	−0.03 [−0.07 to 0.01]	0.13	−0.02 [−0.05 to 0.02]	0.34	−0.03 [−0.08 to 0.01]	0.11	−0.02 [−0.06 to 0.02]	0.32
OD‐fovea angle	0.03 [0.01 to 0.06]	0.02	0.03 [0.01 to 0.05]	0.01	−0.01 [−0.04 to 0.03]	0.75	−0.01 [−0.04 to 0.02]	0.59
Arterial concavity	−0.003 [−0.03 to 0.02]	0.81	0.001 [−0.02 to 0.02]	0.90	−0.02 [−0.05 to 0.01]	0.16	−0.01 [−0.04 to 0.01]	0.31
Venous concavity	0.01 [−0.01 to 0.04]	0.39	0.01 [−0.01 to 0.04]	0.29	−0.02 [−0.05 to 0.02]	0.30	−0.02 [−0.05 to 0.01]	0.30
Vessel tortuosity	0.0004 [−0.03 to 0.03]	0.98	−0.01 [−0.03 to 0.02]	0.58	0.04 [0.002 to 0.07]	0.04	0.03 [−0.004 to 0.06]	0.09
Vessel FD	0.05 [0.01 to 0.09]	0.01	0.02 [−0.01 to 0.06]	0.17	0.05 [0.01 to 0.09]	0.02	0.01 [−0.03 to 0.05]	0.47
CRAE	0.03 [0.01 to 0.06]	0.02	0.02 [0.001 to 0.05]	0.04	0.06 [0.03 to 0.09]	<0.001	0.05 [0.02 to 0.08]	<0.01
CRVE	0.03 [0.01 to 0.06]	0.02	0.02 [−0.01 to 0.04]	0.20	0.05 [0.02 to 0.08]	<0.01	0.03 [−0.01 to 0.06]	0.11
FPI	−0.02 [−0.05 to 0.01]	0.28	0.01 [−0.01 to 0.04]	0.32	−0.02 [−0.05 to 0.02]	0.39	0.01 [−0.02 to 0.05]	0.40

*Note*: A smaller asphericity value indicates a decrease in oblateness or an increase in prolateness.

Abbreviations: CRAE, central retinal arteriolar equivalent; CRVE, central retinal venular equivalent; FD, fractal dimension; FPI, foveal pixel intensity; OD, optic disc; β, standardised beta coefficient.

^a^ Adjusted for spherical equivalent refraction, age and sex.

## DISCUSSION

Most posterior eyes are oblate (even in myopia), but the degree of oblateness decreases as refractive error becomes more negative, with the shape tending towards prolateness in high myopia. A larger angular separation between the OD and fovea is associated with reduced oblateness (or increased prolateness) along the horizontal meridian, independent of refractive error. Central retinal arteriolar narrowing is linked to a similar change in posterior eye shape along both meridians, also independent of refractive error.

The mean posterior eye volume of 4433 mm^3^ (equivalent to 4.433 mL) in the present sample of adults is very similar to the volume (4.650 mL) in another study on adults using computed tomography imaging (noting that the reported unit in the cited study should have been mL),^31^ but is smaller than that reported in a recent MRI‐based study involving children (6350 mm^3^).^5^ The reason for this discrepancy is unclear, but it may stem from the inclusion of additional ocular structures in the latter study because the reported mean volume of 6350 mm^3^ closely matches the mean volume of 6690 mm^3^ found in another investigation that included the lens and anterior chamber.^3^ This discrepancy notwithstanding, previous studies have similarly noted a larger mean volume in males compared with females, with a reported mean difference of 319 and 350 mm^3^ in Azhdam et al.^31^ and Kneepkens et al.,^5^ respectively, which are comparable to the 469 mm^3^ difference found in the present work. The current analysis suggests that this sex difference is attributable to a difference in mean height between males and females (177 vs. 163 cm observed here)—as further adjustment for height eliminates the significant association between sex and posterior eye volume. This is consistent with previous findings suggesting a positive link between body and eye growth.^32^, ^33^

The finding that most posterior eyes conform to a non‐prolate shape, but progressively less so as refractive error becomes more negative, is in general agreement with previous MRI‐based studies.^4^, ^5^, ^7^ For example, in predominantly myopic adults aged 18–36 years, posterior eye shape was found to be oblate in most cases, but decreasingly so and tending towards prolateness in higher myopia.^4^ In a study involving a large cohort of 10‐year‐old children, most eyes were found to have an oblate posterior segment—even in myopic eyes (53% were oblate)—but a higher proportion of eyes with a prolate shape was myopic rather than non‐myopic.^5^

To our knowledge, no prior research has looked at variations in fundus features in relation to differences in MRI‐derived eye shape. The fact that the OD‐fovea angle and CRAE remained significantly associated with posterior eye shape—even after controlling for refractive error—supports the view that some fundus features contain eye shape information because they reflect variations *across* the posterior pole, which are not as well captured by SER alone due to its one‐dimensional, on‐axis nature. Indeed, longitudinal prediction of high myopia onset using machine learning has been found to be more accurate using a combination of baseline SER and fundus photographs as input, rather than relying solely on baseline SER, with the OD and macula being highlighted as important regions for prediction.^34^ In an eye where the posterior segment widens more slowly than it lengthens along the visual axis (decreased horizontal asphericity), the resulting increase in posterior pole curvature (Data S3) along the horizontal meridian would be expected to cause fundus landmarks to appear more horizontally ‘compacted’ when projected onto a two‐dimensional image plane. This might be the reason for the significant association between decreasing horizontal asphericity and increasing OD‐fovea angular separation.

While not statistically significant, other geometrical measures also suggest a more horizontally compacted appearance of the fundus as the posterior eye becomes less oblate (or more prolate) along the horizontal meridian. To illustrate, the SER‐adjusted beta coefficients (Table 3) show a tendency for the temporal artery and vein to curve less inwardly towards the fovea (less concave), coinciding with a decrease in OD‐fovea distance. Conversely, when considering asphericity in the opposite (vertical) meridian, the SER‐adjusted beta coefficients show a tendency in the opposite direction, as a decrease in vertical oblateness (increased prolateness) would cause fundus landmarks to appear more vertically compacted. In contrast to these features, retinal vessel calibre neither described the course of the vessels nor the spatial relationships between fundus landmarks, which possibly explains why the direction of association between CRAE and asphericity remained unchanged regardless of the meridian.

A limitation of this study is the relatively low number of eyes with high ametropia (Figure 5), potentially reducing its generalisability to a broad spectrum of refractive errors. This likely also diminished the overall magnitude of variations in eye shape and fundus features, thus lowering the statistical power of the investigation. Future work including a greater number of eyes with higher ametropia, particularly higher myopia, would be valuable. Additionally, further investigation into whether the association between fundus features and eye shape is similarly independent of axial length would be useful. Another limitation of the present work is that MRI was performed 1.7 years (mean) after the retinal imaging visit. That said, this limitation was mitigated by removing anyone with a time lapse greater than 2 years. Moreover, posterior eye shape is unlikely to change over time in this age group, as eye growth stabilises in young adulthood,^35^, ^36^ and age had no effect on posterior eye volume and asphericity in the present study (Table 2). The effect of head tilt on ocular torsion and, by extension, OD‐fovea angle, may be another limitation. None of the participants, however, had a history of strabismus and the imaging protocol^37^ in the UK Biobank ensured correct positioning of the head and chin. Furthermore, systematic differences in the likelihood and degree of head tilt across eye shape profiles seem unlikely.

## CONCLUSIONS

Variations in OD‐fovea angle and retinal vessel calibre (CRAE) may reflect differences in posterior eye shape, even in eyes with similar refractive error. These features could, therefore, offer additional prognostic value for conditions in which posterior eye shape is clinically relevant. This may be valuable because conventional means of inferring posterior eye or retinal shape (e.g., MRI) are impractical in most settings (e.g., primary care). A promising direction for future research would be to explore if retinal imaging could be leveraged to differentiate the risks of myopia and its associated complications among eyes with *similar* on‐axis refraction (or axial length), since, as demonstrated herein, certain fundus features reflect variations in posterior eye shape even among eyes with similar SER. For example, could retinal information be integrated with (on‐axis) refractive error to derive a more individualised, higher‐dimensional ‘retinal’ equivalent refraction? Such a metric would reflect not only information pertaining to on‐axis refraction but also variations in off‐axis retinal profiles, providing an avenue for personalised risk prediction in clinical practice.

## AUTHOR CONTRIBUTIONS

**Fabian Yii:** Conceptualization (lead); data curation (lead); formal analysis (lead); investigation (lead); methodology (lead); project administration (lead); software (lead); validation (lead); visualization (lead); writing – original draft (lead); writing – review and editing (lead). **Niall C. Strang:** Funding acquisition (equal); supervision (equal); validation (equal); writing – review and editing (supporting). **Samuel Gibbon:** Validation (equal); writing – review and editing (supporting). **Tom J. MacGillivray:** Funding acquisition (equal); supervision (lead); validation (equal); writing – review and editing (supporting).

## FUNDING INFORMATION

Fabian Yii is supported by the Medical Research Council (Grant Number MR/N013166/1). Samuel Gibbon is supported by the Biotechnology and Biological Sciences Research Council (Grant Number BB/M010996/1). The funders had no role in the design or conduct of this work.

## CONFLICT OF INTEREST STATEMENT

The authors declare no competing interests.

## Supporting information


Video S1.

Data S1.

## Data Availability

This study was conducted using data from the UK Biobank under project ID 90655. Data directly supporting the results of this work are only available to the immediate research team members due to UK Biobank's access control policy. Bona fide researchers can, however, apply for access at ukbiobank.ac.uk/enable‐your‐research/apply‐for‐access. Source code used to perform the analyses described in this work is freely available at github.com/fyii200/3DeyeShapeRefractiveError.
