# Supplementary material for: Can fundus features tell us something about 3D eye shape?
Source: Ophthalmic Physiol Opt. 2025 Jan 24;45(4):958–68. doi: 10.1111/opo.13454 (PMC12087834; doi:10.1111/opo.13454)
Supplement: Supplementary file 2 — Data S1. [file OPO-45-958-s002.pdf]

Supplementary Material

S1 Magnification correction

Littmann[1] relates the true size of a dimensional retinal feature, *t*, to its measured size, *s*, as:

$$t = p \times q \times s \quad (1)$$

Where *p* is a variable related to the fundus camera used to image the eye and *q* is a variable dependent on the optical dimensions of the eye being imaged. To derive the ocular factor *q*, Garway-Heath et al.[2] proposed a formula that yielded the most accurate estimate of magnification in the absence of axial length information:

$$q = \frac{1}{(17.21\ /\ \mathrm{CR}) + 1.247 + (\mathrm{SER}\ /\ 17.455)} \quad (2)$$

Where CR represents the corneal radius of curvature (mean of the steepest and flattest meridians was used in this work) and SER represents spherical equivalent refraction.

The camera factor *p* in equation (1) is almost invariably assumed to be a constant in the literature based on the assumption that the fundus camera used to image the eyes is of telecentric construction. However, if the camera is not telecentric, *p* varies from eye to eye depending on the refractive error. An explanation of the principle of telecentricity is available elsewhere.[3, 4] As the Topcon fundus camera used in the UK Biobank cannot be assumed to be telecentric—and information pertaining to *p* is not available for Topcon cameras—we estimated *p* using the average of ten different linear equations derived for ten different non-telecentric camera models, as explained in our recent work:[4]

$$p = 0.015\ \times\ \mathrm{SER} \times 1.521 \quad (3)$$

S2 Image quality control

To minimise noise in the dataset, we implemented strict image quality control for the fundus photographs. Only images with a clearly visible optic disc, fovea and vasculature (arteries & veins) were included. An example of an image deemed suitable for analysis is given below:

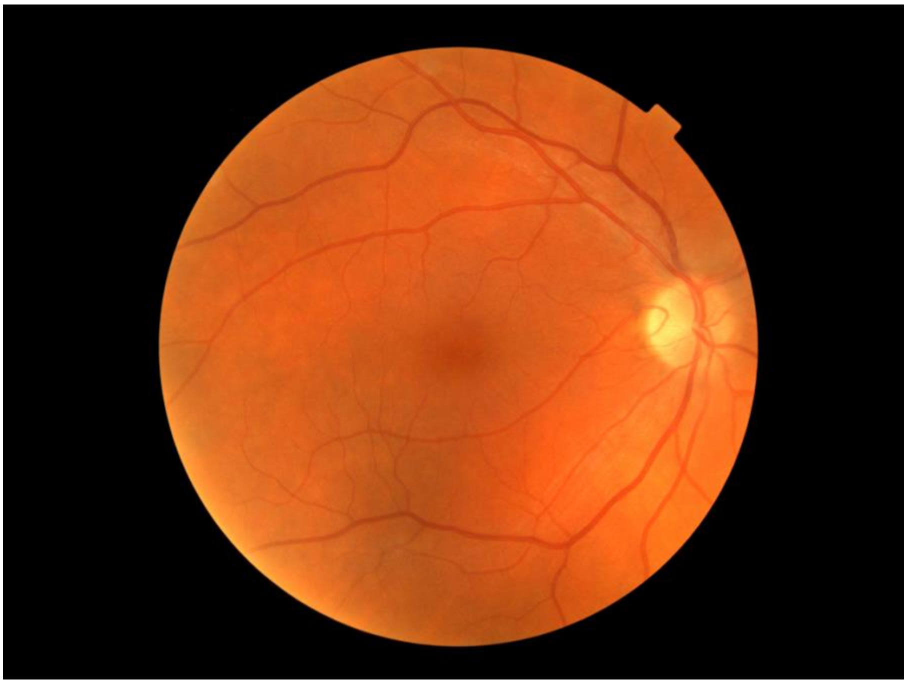

Representative examples of rejected images are provided below, with reasons for exclusion from left to right: (1) general underexposure obscuring both the fovea and inferotemporal vascular arcade, (2) a blink artifact obscuring the inferotemporal vascular arcade, and (3) localised underexposure obscuring both the optic disc and fovea.

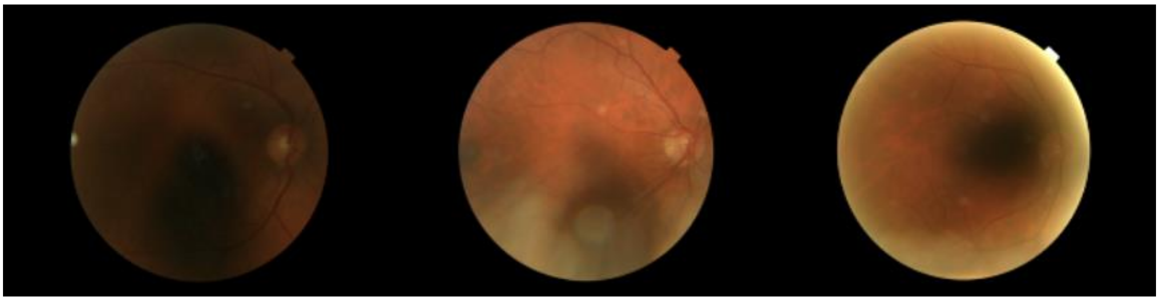

A defacing algorithm was applied to all magnetic resonance imaging scans (MRI) in the UK Biobank to protect participants' anonymity by masking voxels in the face and ear regions (see Alfaro-Almagro et al.[5] for further details). This process often obscured a significant portion of the eye globe, rendering many scans unanalysable for the present study. A representative example of a rejected MRI scan is shown below in both transverse (left) and sagittal (right) orientations:

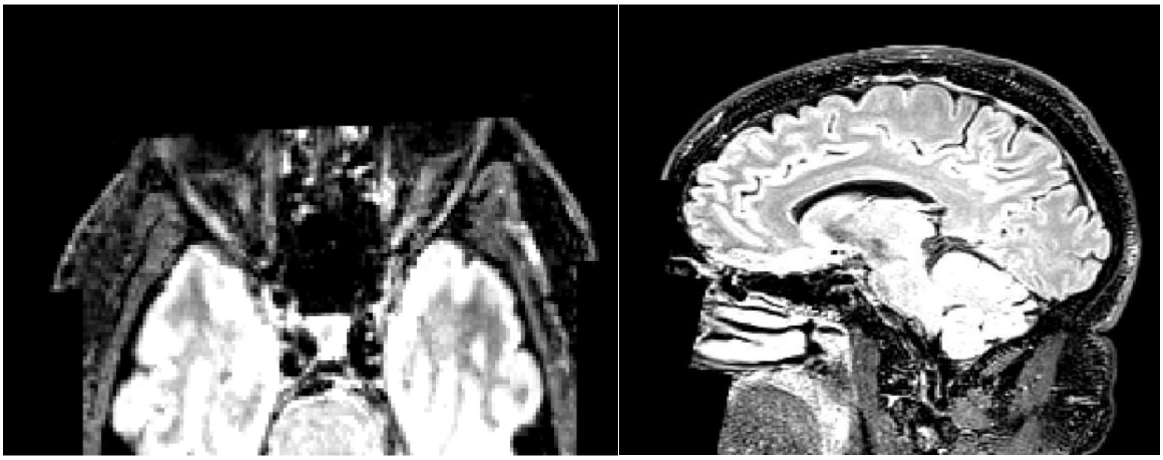

S3 Relationship between asphericity and posterior pole curvature

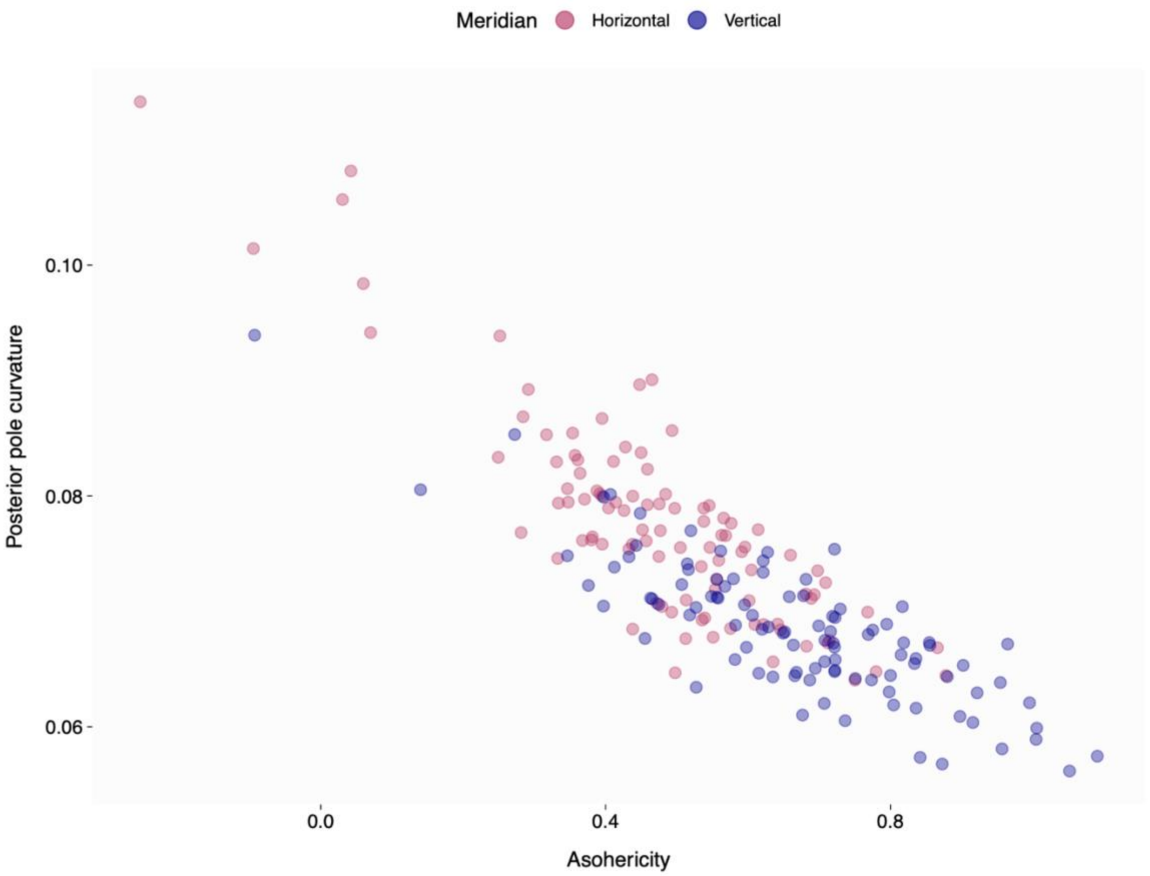

The vertex curvature of the posterior segment of the eye (posterior pole curvature) along the horizontal and vertical meridians can be computed based on the fitted 3D ellipsoid:

$$\textbf{horizontal meridian: } \frac{a}{b^2} \qquad \textbf{vertical meridian: } \frac{a}{c^2}$$

Where a, b and c represent the semi-diameters of the fitted ellipsoid along the anterior-posterior, nasal-temporal and inferior-superior axes, respectively. As shown in the figure above, asphericity is negatively associated with posterior pole curvature (i.e. decreased oblateness or increased prolateness is associated with increased curvature).

References

1. Littmann H. [Determination of the real size of an object on the fundus of the living eye]. Klin Monbl Augenheilkd. 1982;180(4):286-9.
2. Garway-Heath DF, Rudnicka AR, Lowe T, Foster PJ, Fitzke FW, Hitchings RA. Measurement of optic disc size: equivalence of methods to correct for ocular magnification. Br J Ophthalmol. 1998;82(6):643-9.
3. Rudnicka AR, Burk RO, Edgar DF, Fitzke FW. Magnification characteristics of fundus imaging systems. Ophthalmology. 1998;105(12):2186-92.
4. Yii F, Strang N, Moulson C, Dhillon B, Bernabeu MO, MacGillivray T. The optical nature of myopic changes in retinal vessel calibre. Ophthalmology Science. 2024:100631.
5. Alfaro-Almagro F, Jenkinson M, Bangerter NK, Andersson JLR, Griffanti L, Douaud G, et al. Image processing and Quality Control for the first 10,000 brain imaging datasets from UK Biobank. Neuroimage. 2018;166:400-24. Epub 20171024.
